# Hemorrhoids with Scirrhous Rectum

**Published:** 1850-05

**Authors:** R. Willard

**Affiliations:** Oswego, Ind.


### ARTICLE II.

*Hæmorrhoides with Scirrhous Rectum*.—By R. WILLARD, M.D. of Oswego, Ind.

Mr. Alex. C. Bull, æt. 26, of good constitution, and temperate habits, was seized with a disease of the anus, supposed to be Piles, at the age of 20 or 21; after becoming troublesome, was treated by Physicians in Massilon and Wooster, Ohio, but without any apparent benefit. In 1846 he removed to Indiana, so infirm as not to be able to labour, nor had he been for a year previous to his return to his friends here. The diseased state of the anus and lower portion of the rectum, was becoming more and more formidable, and he resolved to visit Cincinnati, and put himself under the care of Messrs. Hill & Morrow, the former, Professor of Surgery in the Eclectic Medical School. These gentlemen excised by ligature one tumor; cauterized some, and discharged him with an assurance that he would soon be well. What the bill of these gentlemen was, I did not learn, but the brother informed me that during his stay of nine weeks, it cost him $400. Suffice it to say, that he returned to his friends unbenefitted. Science having failed to restore him ; he again applied to some German pretender, and then to a professed Botanic, and finally, when the symptoms became so urgent as to threaten the citadel of life, I was sent foı also, and found him in the following condition: When repairing to his bed side, I asked him how he did. With apparent emotions of hope and despair alternating, he turned down the clothes to show me what a dreadful state he was in. My senses were shocked on beholding the enormous size of his abdomen, which I found on examination, to be tympanitic, and greatly emaciated ; with hectic pulse ; flushed face ; parched tongue ; dyspnœa, and much restlessness ; wanted water constantly, and as frequently ejected it.

Having obtained a succinct history of his case, and being informed that he had not for some 86 or 88 days, had what might be considered an operation from the bowels, I proposed to examine the nature of the difficulty. He was accordingly laid upon a low bed, near the window, for examination. The sphincter ani, rectum, and all the soft parts between the tubera ischii, appeared to be a perfect mass of Scirrhus, and to the touch quite unyielding. I then attempted to introduce a metalic sound of some nine inches in length, feeling and probing into several fissures, and ultimately succeeded in passing the instrument up the rectum. On its withdrawal, no feces followed. I then informed him and his friends, that I saw no way in which I could render him a permanent benefit, and that a reduction of the abdomen was absolutely necessary to any relief, and in order to effect such a result, I knew of no other way than to discharge the gas by puncture of the parietes of the abdomen; but viewing the case hopeless, I should not attempt the measure without the full and entire consent of the patient and his friends; the latter of whom made no objections, while the patient, conscious that something must be done, wished me to operate. Having with me a small trocar, I passed it into the abdomen through the linea alba, some two inches below the umbilicus; the effect was, on withdrawing the stilet, that a sudden and forcible escape of gas followed, and the abdomen was reduced to nearly an ordinary size. The patient made no complaint in the operation, and said he felt relieved. My mind was now drawn to a consideration of the cause of this untoward phenomenon, as regarded the nature and extent of the disease, as also the practicability of any permanent relief. It occurred to me that possibly the natural passage might be made pervious, or an artificial one substituted. I therefore ordered an elder quill with the pith removed which I dressed up with cotton flannel in a manner so as not to lacerate the parts.

Having prepared a mucilage of cortex ulmi and lubricated my finger, with cautious perseverance I succeeded in passing it down the scirrhous affection in the channel of the true rectum; but the indurated condition of the surrounding parts, were so resisting as to preclude any passage of feces. Having withdrawn the finger, I then attempted the introduction of the canula, which, after using some address, turning and twisting, was carried above the stricture. The result was a passage of dark colored feces, in the amount and consistence of something more than an ordinary discharge from physic. About this time a sensible chill came over him, with returning pains and fulness of the bowels. Having taken a saucer of gruel soon after the operation of paracentesis, the question arose in my mind, whether it had become fermented so as to produce the tympanitic state of the abdomen, or whether a putrescent state of the viscera had not produced it. Suffice it to say he soon became inflated; while a train of symptoms began to assure me that death would claim its victim. He died in about four hours from the time I first saw him.

Now, the inquiry will arise, can the anus and rectum be so diseased as to utterly preclude fœcal discharge? Can a person subsist three months, by what chylopoietic nutrition the stomach and upper bowels are able to concoct, and take up, and at the same time the lower bowels serve as a reservoir for the feces during that time? Such seems to have been the above case, though the patient was greatly atrophied. Had it been practicable, it would have been interesting to examine into the pathological condition in the above case, and thereupon premise some efficient method of treatment; but circumstances did not allow a post mortem examination to be made.

Should the above case be thought of sufficient interest to find a place in your journal, it is at your option to insert or otherwise.

